# Establishing the Trial Ready Registry and K‐Dementia to enhance Alzheimer's disease research in Korea

**DOI:** 10.1002/alz70855_102546

**Published:** 2025-12-23

**Authors:** Inhee Mook‐Jung

**Affiliations:** ^1^ Korea Dementia Research Center, Seoul, Korea, Republic of (South); Seoul National University, College of Medicine, Seoul, Korea, Republic of (South)

## Abstract

**Background:**

Numerous studies on Alzheimer's disease (AD) are currently underway, and Korea possesses a wealth of high‐quality clinical data and resources on AD. However, the lack of thorough standardization has made these resources difficult to utilize effectively. To address this, the Trial Ready Registry (TRR) and K‐Dementia platform were established to identify potential therapeutic targets and biomarkers for AD. These initiatives aim to advance the AD research field in Korea by standardizing and integrating dementia‐related datasets.

**Method:**

To collect human‐derived materials, we developed the Minimum Common Dataset (MCD), which includes clinical evaluations, cognitive assessments, magnetic resonance imaging (MRI), Florbetaben (FBB)‐PET, Flutemetamol (FMM)‐PET, and blood tests. Dementia data from both prospective and retrospective cohorts were collected using a standardized model based on MCD and SNOMED‐CT. A centralized database system was created to manage data storage, exchange, and integration. This system supports comprehensive data analysis, including visualization, statistical evaluation, and deep learning, to enhance data usability.

**Result:**

The TRR‐DPK system aims to recruit 3,000 participants for MCD evaluations, blood tests, and brain imaging by 2028. As of December 2024, over 2,000 participants have been enrolled in the TRR since its launch in 2020. Of the target participants, approximately 1,800 will undergo longitudinal follow‐up every two years for additional data and blood sample collection. The K‐Dementia platform has retrospectively secured over 10,000 datasets from Korea and established a database schema informed by global dementia databases. Additionally, a cloud‐based system integrated with AWS was developed to enable secure data analysis, allowing researchers to perform analyses in a protected environment where only the results can be downloaded.

**Conclusion:**

The TRR and K‐Dementia platform are expected to significantly advance AD research by providing researchers with access to high‐quality, human‐derived materials and standardized datasets. Furthermore, these initiatives will foster global collaboration, particularly with platforms such as the Dementias Platform UK (DPUK), through the development of standardized data and secure analytical environments.

